# Reviving Matrix for Nerve Reconstruction in Rabbit Model of Chronic Peripheral Nerve Injury With Massive Loss Defect

**DOI:** 10.3389/fsurg.2020.609638

**Published:** 2021-01-15

**Authors:** Shimon Rochkind, Mara Almog, Sigal Meilin, Zvi Nevo

**Affiliations:** ^1^Research Center for Nerve Reconstruction, Tel Aviv Sourasky Medical Center, Sackler School of Medicine, Tel Aviv University, Tel Aviv, Israel; ^2^Neurology R&D Division, MD Biosciences, Ness Ziona, Israel; ^3^Department of Human Molecular Genetics and Biochemistry, Sackler School of Medicine, Tel Aviv University, Tel Aviv, Israel

**Keywords:** antigliotic guiding regenerative gel, artificial peripheral nerve, peripheral nerve injury, guiding regeneration gel, nerve regeneration

## Abstract

**Background and Aims:** The aim of this study was to investigate the innovative guiding regenerative gel (GRG) and antigliotic GRG (AGRG) fillings for nerve conduits, prepared with Food and Drug Administration (FDA)-approved agents and expected to provide an alternative to autologous nerve graft and to enable reconnection of massive nerve gaps in a rabbit model of chronic peripheral nerve injury with massive loss defect that simulates the human condition of chronic injury with a large gap.

**Methods:** The components and dosimetry for GRG and AGRG formulations were investigated *in vitro* on nerve cell culture and *in vivo* on 10-mm reconstructed sciatic nerves of 72 rats using different concentrations of agents and completed on a rabbit model of delayed (chronic) complete peripheral nerve injury with a 25-mm gap. Forty rabbits underwent delayed (9 weeks after complete injury of the tibial portion of the sciatic nerve) nerve tube reconstruction of a gap that is 25 mm long. GRG and AGRG groups were compared with autologous and empty tube reconstructed groups. Rats and rabbits underwent electrophysiological and histochemical assessments (19 weeks for rats and 40 weeks for rabbits).

**Results:** Application of AGRG showed a significant increase of about 78% in neurite length per cell and was shown to have the most promising effect on neuronal outgrowth, with total number of neurites increasing by 4-fold. The electrophysiological follow-up showed that AGRG treatment is most promising for the reconstruction of the tibial portion of the sciatic nerve with a critical gap of 25 mm. The beneficial effect of AGRG was found when compared with the autologous nerve graft reconstruction. Thirty-one weeks post the second surgery (delayed reconstruction), histochemical observation showed significant regeneration after using AGRG neurogel, compared with the empty tube, and succeeded in significantly regenerating the nerve, as well as the autologous nerve graft, which was almost similar to a healthy nerve.

**Conclusion:** We demonstrate that in the model of delayed peripheral nerve repair with massive loss defect, the application of AGRG led to a stronger nerve recovery and can be an alternative to autologous nerve graft.

## Introduction

Peripheral nerve injury (PNI) occurs in about 2.8% of all trauma patients and can cause disability and a significant decrease in quality of life (1). A growing number of traffic and work accidents, natural disasters, and military activity often result in PNIs, causing lifelong dysfunction associated with loss of sensory and motor functions, and in some cases intractable pain, and requiring long-term peripheral nerve rehabilitation treatments. There are about 300,000 cases of PNIs per year (2). The annual incidence rate of nerve injuries is reported to be 13.9/100,000 inhabitants per year (3). In the USA alone, 50,000 nerve graft procedures are performed annually (4), accounting for seven billion USD in expenses. This indicates that improved treatment strategies for PNIs may not only improve the situation for the patients but also significantly reduce costs for the society.

The current standard of care for PNIs includes the gold standard autografts, Food and Drug Administration (FDA)-approved hollow conduits, and decellularized nerve allografts (5). The gold standard for the reconstruction of nerve damage is non-immunogenic nerve grafts (6) that have been harvested from the same patient. Depending on the extent of the nerve injury or the distance to overcome, a complete reconstruction can be difficult or even impossible due to the limited extent of the grafting material. Furthermore, autologous nerve graft (ANG) may result in a painful neuroma formation at the donor site with loss of the donor nerve function (7).

Nerve guidance conduits represent a biomaterial-based scaffolding to aid in nerve repair and regeneration to bridge nerve defects and guide axon regeneration to the appropriate distal target. Currently, there are 11 FDA-approved conduits for treatment of PNI produced with biomaterials, of both natural and synthetic origins. The advantages of the nerve guiding conduit in comparison to the ANG are the simplicity of the procedure, a significant decrease in time of surgery, and no sensation loss or cosmetic defect as a result of donor site intervention. On the other hand, the main disadvantage of the nerve guiding conduit is the inability to bridge nerve loss that is more than 2–2.5 cm long.

Guiding regenerative gel (GRG), as previously reported by Rochkind and Nevo (8), was developed with the aim of enabling reconstruction of injured peripheral nerve with massive loss defect by using commercial nerve guiding conduits. GRG is a special milieu that increases nerve growth and promotes recovery, aiming, ultimately, at restoring the function of an affected nerve. The major advantages of the GRG lay in its composition, including the three most important and essential elements needed in the initial period of the adjustment and integration of the implant in its new surrounding: (1) antioxidants, found to exhibit high anti-inflammatory activities; (2) synthetic laminin peptides, which act as a scaffold for the nerve fibers to grow along; and (3) hyaluronic acid (HA), which is highly hydrated and contributes to the success of survival, growth, and regeneration of nerve fibers by protecting them from drying.

An *in vivo* study (3 months) on peripheral nerves with massive nerve loss showed that GRG loaded into a commercial collagen tube enabled massive growth of myelinated axons and continuation of axonal sprouting through the tube to the distal part of the nerve in a 15-mm-long gap in the sciatic nerve in rats, which is not possible when bridging with an empty tube. No significant difference was found between GRG and the “gold standard” treatment (nerve autograft) study groups, emphasizing that the GRG enables optimal axonal regeneration. In an additional functional study (9), we evaluated the efficacy of GRG in restoring function to paralyzed limb following a massive nerve loss defect of 15 mm. Three groups were studied: ANG and an implantation of empty tube, with and without GRG. After 6 months of follow-up, we found the group with tubes filled with GRG to be superior to the current gold standard treatment by its ability to regain function, where an empty tube was unable to support any movement.

While the rat model remains the first choice for *in-vivo* testing, it has been postulated that the disproportionate number of studies using rats may in fact skew treatment outcomes and lead to inappropriate evaluation of risks and benefits (10). One example of why a larger animal model may need to be chosen is the limit in nerve gap length that can be studied. While the rat model has effectively been used for short nerve gap model, nerve regeneration over longer gap lengths is far more challenging, with the mode of reconstruction playing a determining factor in recovery (11, 12). This is especially true as the critical gap length for humans of near 3 cm is reached. As such, larger animal models have become more widely considered and further examined for clinical translations, especially when gaps longer than 1.5 cm (13–15) are being evaluated. Of these, a rabbit model is most widely used. To date the sciatic nerve injury is the most well-documented nerve injury model in rabbits with 45% of studies carried out using the sciatic nerve (13–15). There are distinct advantages to utilizing rabbits as the chosen animal model (16). Furthermore, using the rabbit for a peripheral nerve model has allowed for the testing of injuries more than 2 cm, with documented cases on the facial, sciatic, peroneal, median, radial, and ulnar nerve (17, 18).

The aim of this study was to investigate the modified GRG and new combination of AGRG fillings for nerve conduits, prepared with FDA-approved agents, and expected to provide an alternative to an ANG, by supporting and enhancing axonal regeneration, enabling reconnecting massive nerve gaps. The components and dosimetry for new GRG and AGRG formulations were investigate on *in-vitro* nerve cell culture, *in-vivo* rats model and completed on rabbit model of the delayed (chronic) PNI with massive loss defect that represents the human condition (chronic, large gap).

## Materials and Methods

### *In vitro* Study—Spine Primary Culture

One-month Sprague-Dawley rats were euthanized anesthetized with Ketamine-xylazine solution (100 and 10 mg/kg, respectively). Then, the spinal cord was removed, placed in a sterile 10 mm petri dish with Hank's Balanced Salt Solution (HBSS) medium (without calcium and magnesium; Biological Industries Ltd., Israel) buffered with 2% HEPES (Biological Industries Ltd., Israel), and kept on ice. The meninges were stripped away, and the spine was dissected into small pieces and collected into a 15 ml centrifuge tube with 40% TrypLE (Biological Industries Ltd Israel), in HBSS. The tube was agitated horizontally at room temperature for 20 min. Then, the tube was centrifuged for 5 min at 200 g. The supernatant was discarded and 2 ml of fresh Complete Culture Media (Biological Industries Ltd., Israel) were added. The cells were dissociated by pipetting up and down 10 times, first in a normal Pasteur pipette, and then 10 times in a pipette with a tip fire polished to nearly half the normal diameter. Clumps were left to stand for 5 min and then the supernatant was collected into a new 15 ml centrifuge tube. The cells were then seeded on a coverslip in Complete Culture Media (containing 5% horse Serum; Biological Industries Ltd., Israel) for 24 h. After 1 day, the Complete Culture Media was replaced to Incomplete Culture Media (Biological Industries Ltd., Israel), containing Dulbecco's Modified Eagle Medium (DMEM; Biological Industries Ltd., Israel) with 2% B27 (Rhenium, Israel) and 1% Glutamax (Rhenium, Israel), and the tested compounds were added and the study was finalized at day 10. All assays were run in triplicated and repeated at least twice.

### Preparation of GRG/AGRG

A stock solution of 25 ml/mg synthetic laminin peptide consisting of 16 amino acids (synthesized at Bachem, Switzerland) was aseptically prepared by diluting the laminin in 100% dimethyl sulfoxide (DMSO; Sigma-Aldrich), filtered with 0.22-μm filter, and divided into aliquots, and stored at −20°C. For a final concentration of 10 μg/ml, the stock solution was diluted in DMSO to obtain a solution at a concentration of 5 mg/ml. For the *in vitro* studies, 4 μl of the solution was added to 2 ml of incomplete culture media; for the *in vivo* studies, 4 μl of the solution was added to 2 ml of phosphate-buffered saline (PBS; Biological Industries Ltd., Israel).

dl-α-Tocopherol (Merck Millipore, Israel) was diluted with 1 ml of 100% DMSO, generating a stock solution at a concentration of 450 mM with 60% DMSO. The stock solution was used to achieve the different tocopherol concentrations used in the experiments (for 10 and 3 mM, the stock solution was used; for 1 mM, the stock solution was diluted 1:3 with 60% DMSO to achieve a solution at a concentration of 150 mM; for 0.3 mM, the stock solution was diluted 1:10 with 60% DMSO, giving a concentration of 45 mM). Then, to reach a final concentration of 0.3, 1, and 3 mM, 6.67 μl of each solution was added to every 1 ml of either culture medium (for the *in vitro* studies) or PBS (for the *in vivo* studies). To reach a final concentration of 10 mM, 22.23 μl of the stock solution was added to every 1 ml of PBS (for the *in vivo* studies).

HA (0.4%) of high molecular weight (1.67 MDa) (Lifecore Biomedical, USA) was prepared aseptically with incomplete culture media for the *in vitro* studies and with PBS for the *in vivo* studies and stored at 4°C.

The final GRG formulation for the *in vivo* rat study was prepared as 0.4% HA solution with the addition of laminin at a final concentration of 10 μg/ml and tocopherol at final concentrations of 0.1, 1, 3, and 10 mM. The final DMSO concentration was about 0.6%.

Additionally, 10 μg/ml of Copaxone (glatiramer acetate; Teva Pharmaceutical Industries Ltd.) was added to the GRG hydrogel to test an additional benefit in neuronal outgrowth. A stock solution of 20 mg/ml was used, and 0.5 μl was dissolved either with 1 ml of medium in the *in vitro* study or with 1 ml of PBS in the *in vivo* study, to reach a final concentration of 10 μg/ml.

The final AGRG formulation for the *in vivo* rabbit study was prepared as 0.4% HA solution with the addition of laminin at a final concentration of 10 μg/ml, tocopherol at a final concentration of 3 mM, and Copaxone at a final concentration of 10 μg/ml. The final DMSO concentration was about 0.6%.

### *In vitro* Analyses

A full scan and imaging of three coverslips per treatment group were taken. Imaging was done using a BX43 Olympus microscope driven by the standard “CellSens” software by Olympus. Images were taken under 20X objective using a DP74 camera (Olympus). To estimate the neurite length, an ImageJ plugin—“NeuronJ”—was used. Pictures from different areas were taken at various time points from at least three wells per treatment group. The following readouts were measured using ImageJ software with the NeuronJ plugin: (1) mean neurite length per cell, total number of neurites; (2) total number of cells; (3) total number of neurites, mean neurite length per cell; (4) mean number of neurites per cell; and (5) mean number of bifurcations per cell.

### Animals and Surgical Procedure

All animal experiments were approved by the Council for Experiments of Animal Subjects at the Israeli Ministry of Health and adhered strictly to the Animal Care guidelines. The animals were housed under standard conditions [room temperature 20–24°C; a relative humidity (RH) of 30–70%; a 12:12 h light:dark cycle; 15–30 air changes per hour in the study room]. Food and water were provided *ad libitum*.

#### Rat Acute PNI Model

Seventy-two male Wistar rats, weighing 250–300 g, were anesthetized using an intraperitoneal injection of 10% ketamine (35 mg/kg) and 2% xylazine (8 mg/kg) mixture. Then the animals were placed on the surgery table. The area of the surgery was shaved, washed with ethanol and Polydine solution, and then covered with a sterile sheet to ensure sterile conditions. The operation on the sciatic nerve was carried out on the left hind limb. Rats were put in a prone position, with the hind limbs abducted, and the skin over the lateral and caudal aspects of the limb up to the lumbar midline was sheared. An incision of about 4–5 cm in length was made along the fusion line of the muscles. The fascia was sharply divided, and the muscles were bluntly retracted to enable access to the sciatic nerve. With a microscope, the sciatic nerve was exposed and was transected proximally and distally, removing 10 mm of length using a microsurgical razor. Prior to transection closure, Marcaine 0.5% (Vetmarket, Israel) was applied. All groups underwent neural reconstruction with either ANG or a NeuraGen® tube (Integra LifeSciences, USA) (the groups are described in Table 1). Then the nerve was reconstructed as follows:

1. *ANG* (group 1): The removed 10-mm nerve segment was inverted and implanted between proximal and distal parts of the nerve. Immediately afterwards, an end-to-end anastomosis was performed between the peripheral nerve segment and the proximal and distal parts of the left sciatic nerve, using 10-0 sutures. Cooptation of the nerve was carried out in order to preserve all of the fascicles within the epineural sac. The muscles were sutured using 3-0 Vicryl threads. The skin was closed using special metal staples.
2. *NeuraGen*^®^ *Nerve Guide tube* (groups 2–5): After removal of the 10-mm nerve segment, the proximal and distal ends of the nerve were fixed into the 15-mm NeuraGen® Nerve Guide tube (Integra LifeSciences, USA) pre-immersed in saline, creating a 10-mm gap between the two ends, and were microsurgically reconnected using 10-0 epineural sutures. In groups 3–5, before the second end of the nerve was sutured, the corresponding GRG treatment was injected into the NeuraGen® Nerve Guide (see Table 1). The external connective area between the tube and the nerve was covered by TISSEEL sealant (Baxter, USA). Then the muscles were sutured using 3-0 Vicryl threads, and the skin was closed using special metal staples.

**Table 1:** Rat acute PNI experimental design.

**Treatment**	***n***
1. Autologous nerve graft (ANG)	12
2. NeuraGen® Nerve Guide	12
3. NeuraGen® Nerve Guide filled with GRG (0.3 mM tocopherol)	12
4. NeuraGen® Nerve Guide filled with GRG (1 mM tocopherol)	12
5. NeuraGen® Nerve Guide filled with GRG (3 mM tocopherol)	12
6. NeuraGen® Nerve Guide filled with GRG (10 mM tocopherol)	12

#### Rabbit Chronic PNI Model

##### Induction of PNI (First Surgery)

Forty-one female New Zealand White rabbits, weighing 2.5–3 kg, were anesthetized using intramuscular injection of 10% ketamine (35 mg/kg) and 2% xylazine (5 mg/kg) mixture. Then, the rabbits were placed on the surgery table and connected to an anesthetic machine that delivered isoflurane (1.5–3%) and 100 oxygen mixture at a rate of 0.5–15 L/min. The area of the surgery was shaved, washed with ethanol and Polydine solution, and then covered with a sterile sheet to ensure sterile conditions.

The operation on the tibial portion of the sciatic nerve was carried out on the left hind limb. The rabbit was put in a prone position, with the hind limbs abducted, and the skin over the lateral and caudal aspects of the limb up to the lumbar midline was sheared. An incision of about 7 cm in length was made along the fusion line of the muscles. The fascia was sharply divided, and the two muscles (biceps femoris and semimembranosus) were bluntly retracted to enable access to the sciatic, peroneal, and tibial nerves. With a microscope, the tibial portion of the sciatic nerve was exposed. Marcaine 0.5% (Vetmarket, Israel) at a volume of 100 μl at each side of the nerve was applied epineurally to the dissected area. The tibial nerve was transected proximally and distally removed at 1 cm of its length. The ends of the transected nerve were sutured to the muscle to prevent possible sprouting of axons. Then the muscles were sutured using 3-0 Vicryl threads, and the skin was closed using special metal staples.

##### Repair of the PNI (Second Surgery)

Nine weeks after the induction of the injury, 40 rabbits were re-anesthetized (one rabbit was culled after the first surgery due to ethical reasons; see Table 2), as described in the first surgery, and the initial PNI was repaired. All groups underwent neural reconstruction with either ANG or a NeuraGen® tube (Integra LifeSciences, USA) (Table 2). Then the nerve was reconstructed as follows:

1. ANG (group 1): The right hind limb and the left hind limb were shaved, cleaned with soap and water, and then washed with ethanol and Polydine solution. With a microscope, the right tibial portion of the sciatic nerve was exposed. Marcaine 0.5% at a volume of 100 μl at each side of the nerve was applied to the dissected area. A tibial nerve segment of 2.5 cm was extracted using a microsurgical razor. Then, the muscles were sutured using 3-0 Vicryl threads, and the skin was closed using metal staples. After that, the 2.5-cm piece of the right tibial nerve was reversed and transplanted to the left limb, after exposing the transected tibial nerve in that limb. The ends of the previously transected nerve of the left hind limb were released, and a 4 mm portion from the proximal and distal ends was removed. Immediately thereafter, an end-to-end anastomosis with a 2.5-cm autologous graft was performed between the proximal and distal parts of the left tibial nerve, using 10-0 sutures. Cooptation of the nerve fascicles was carried out in order to preserve all the fascicles within the epineural sac. Then the muscles were sutured using 3-0 Vicryl threads. The skin was closed using metal staples.
2. NeuraGen® Nerve Guide tube (groups 2–4): The left hind limb was shaved, cleaned with soap and water, and then washed with ethanol and Polydine solution. The operation was carried out by exposing the proximal and distal ends of the left tibial nerve and separating it from the muscles. The transected tibial nerve ends were released, and a 4-mm portion from each of the transected end was removed. The proximal and distal ends of the nerve, 2.5 mm each, were fixed into 3 cm of the NeuraGen® Nerve Guide tube, creating a 2.5-cm gap between the two ends, and microsurgically reconnected using 10-0 epineural sutures. In groups 3 and 4, before the second end of the nerve was sutured, the corresponding GRG/AGRG treatment was injected into the NeuraGen® Nerve Guide (see Table 2). The external connective area between the tube and the nerve was covered by TISSEEL sealant (Baxter, USA). Then the muscles were sutured using 3-0 Vicryl threads, and the skin was closed using metal staples.

**Table 2:** Rabbit chronic PNI experimental design.

**Treatment**	**1^**st**^ surgery**	**2^**nd**^ surgery**
1. Autologous nerve graft (ANG)^*^	8	8
2. NeuraGen® Nerve Guide	11	10
3. NeuraGen® Nerve Guide filled with GRG	11	11
4. NeuraGen® Nerve Guide filled with AGRG	11	11

* *This group was conducted separately, as part of a developmental experiment, but the experiment design was the same as in the efficacy experiments (groups 2–4)*.

### Electrophysiological Assessment

Non-invasive electrophysiological evaluation was performed before the surgical procedure and again between surgeries in the chronic PNI model and following the repair PNI surgery (the second surgery in the chronic PNI model). The anesthetized animals were placed in a prone position on a heating pad that was only switched off for the short period of actual recording to keep their body temperature at ≤36.5°C. In the chronic PNI model, the rabbits were then connected to an anesthetic machine that delivered oxygen at a rate of 0.5–15 L/min. The recordings were performed in the operated left limb and the right limb using a Dantec® Keypoint® focus device (Natus Medical Inc., USA). Bipolar stimulating needle electrodes were placed at the sciatic notch and the paired recording needles at the gastrocnemius muscle in the rabbits and in the tibialis anterior muscle in the rats. The ground electrode was placed on the thigh on the side of stimulation. The sciatic nerve was stimulated by a bipolar stimulating electrode with a pulse of 0.1-ms duration. The stimulus intensity was increased gradually, up to 30% supramaximal level. Then, evoked compound muscle action potentials (CMAPs) were recorded. CMAP amplitude (baseline to negative peak of the M-wave) was measured and normalized to the value measured between the surgeries (chronic PNI model) and to the value measured at baseline (acute PNI model). In cases when animals did not show a CMAP, the amplitude was set to 0.

### Histological and Immunohistochemical Evaluation

Thirty one weeks post the second surgery, the tibial portion of the sciatic nerve was harvested and cross-sectioned into three pieces: proximal to the injury, middle (the injury area), and distal to the injury. The tissues were fixed in 10% formalin and processed and embedded in paraffin blocks. Finally, 132 paraffin blocks of the tibial portion of the sciatic nerve of 40 animals were evaluated (12 paraffin blocks from the healthy right hind of four rabbits).

Embedded tissues in paraffin blocks were sectioned at ~5-μm thickness, two slides per block; put on a glass slide; and stained with hematoxylin and eosin (H&E, Rhenium, Israel) and immunohistochemistry with myelin basic protein (IHC:MBP, Zotal, Israel). The stained slides were subjected to histological evaluation. Then, pictures were taken using a microscope (Olympus BX60, serial no. 7D04032) at a magnification of X4 with the microscope's camera (Olympus DP73, serial no. OH05504). Picture acquisition was performed only on pathological changes and of representative animals. Image analysis was done with the Image Pro Plus version 6.3 software (Media Cybernetics, USA). An area of interest (AOI) and spatial calibration were applied to each image. Then an RGB histogram threshold was used to depict the brown stain, and the area and area ratio (%) of each threshold were measured.

We performed two stains separately: H&E to assess the quality of the sample and IHC:MBP to evaluate the number of intact motor fibers, neuron fibers, and myelination. Representative pictures were taken by a pathologist. The relative areas of myelin fibers were calculated using the digital morphometric method with IHC:MBP-stained samples (means ± SEM of the different groups were calculated).

### Statistical Analyses

GraphPad Prism version 6.07 (GraphPad Software, USA) was used to perform statistical analyses of the data recorded in this study. To detect significant differences, one-way ANOVA followed by Tukey's multiple comparisons, one-way ANOVA followed by Holms test, and one-way ANOVA followed by Dunnett's multiple comparisons (electrophysiological assessment and *in vitro* study) were applied. For the immunohistochemistry analyses, one-tailed and two-tailed Student's *T*-tests were applied. The *p* value for statistical significance was set to *p* < 0.01, *p* < 0.05, or *p* < 0.01. All results are presented as percentages or mean ± SEM indicated in the respective tables or figures. For the statistical analyses of electrophysiological evaluation (CMAP amplitude) and immunohistochemistry analyses, animals had to be excluded due to ethical reasons.

## Results

### *In vitro* Study

The *in vitro* studies were conducted to show the effect of the GRG and AGRG on the neuronal outgrowth. For this study we used a similar GRG formula, as we previously reported (8), but we decided to substitute the antioxidant substance (superoxide dismutase 1; SOD1) to a substance that is clinically approved. Thus, we conducted an *in vitro* study to find the best antioxidant substance and it concentration. Following this experiment, we chose tocopherol at a concentration of 3 mM to substitute the SOD1 in the GRG formula, since it showed the best outgrowth in the spine primary neurons (data not shown). Afterwards, we conducted an additional *in vitro* study to evaluate the effect of several compounds, each in combination with GRG (i.e., AGRG) to study the neurite outgrowth in spinal primary neurons of adult rat. Treatment with GRG+copaxone 10 μg/ml showed to most promising effect on the neuronal outgrowth (data not shown).

Due to the used of DMSO to solve some of the compounds in the GRG formula (see method and materials), we performed a control *in vitro* study to test the effect of 1% DMSO, which is the highest concentration used, on neurite outgrowth of the spine primary neurons. No significant change in the mean neurite length per cell was seen following the addition of 1% DMSO (data not shown).

Consequently, the AGRG formula contains HA 0.4%, tocopherol 3 mM, laminin 10 μg/ml and copaxone 10 μg/ml.

**Figure 1:**
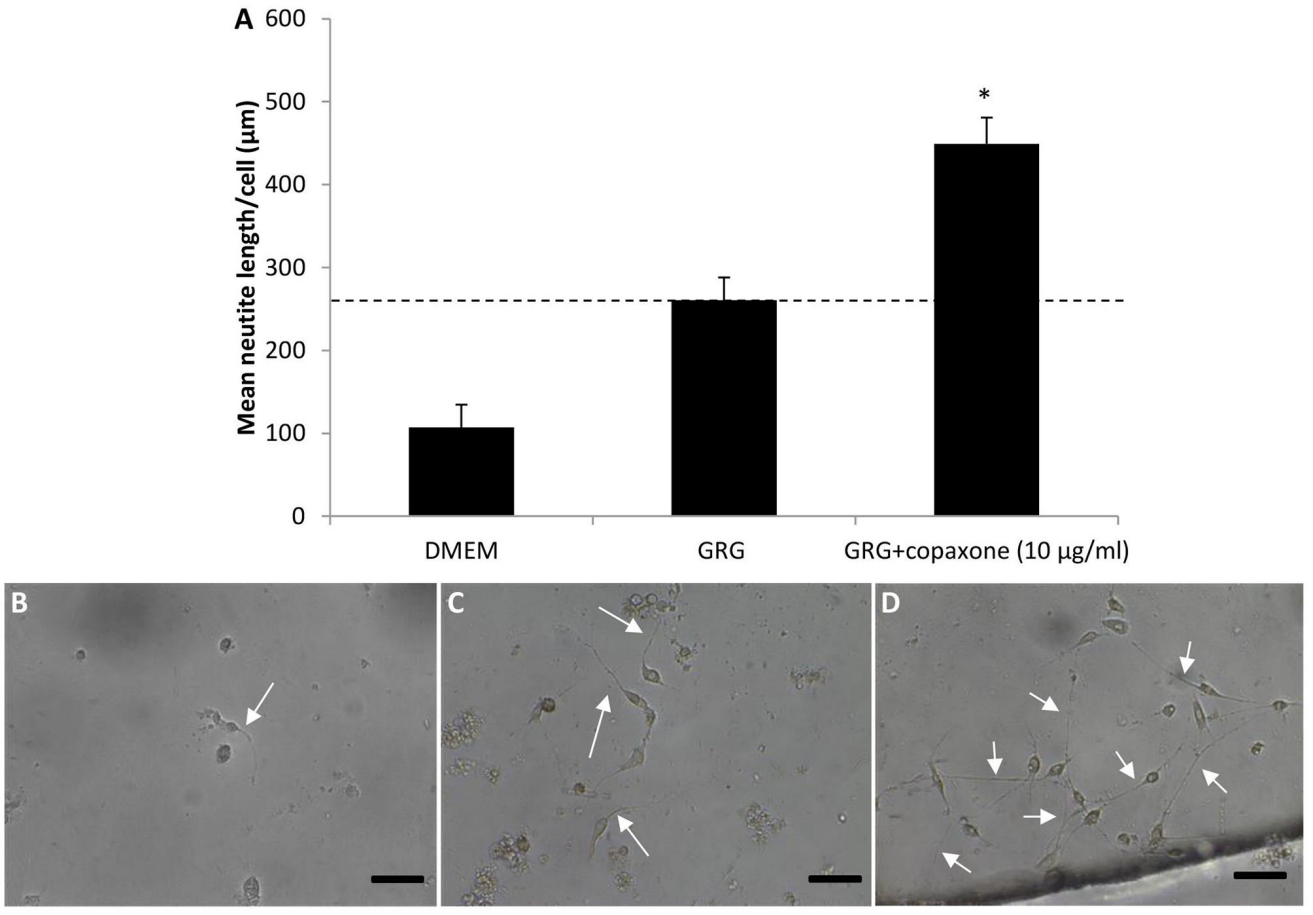
The effect of different formulation of AGRG on neurite outgrowth. **(A)** The bars displays the mean neurite length per cell in um. Results are presented as mean ± SEM. Asterisk represents statistical significance: *p < 0.05 vs. GRG, using one-way ANOVA followed by Dunnet's test. **(B–D)** Representative pictures of the spinal primary cultures treated with DMEM, GRG and GRG+copaxone are displayed. White arrows indicate the neurite outgrowth. The scale bas displayed represents 50 μm.

Figure 1 and Table 3 show that treatment with GRG resulted in an increase of more than 50% in neurite outgrowth vs. DMED-treated cells. Adding 10 μg/ml of Copaxone to the GRG (AGRG) resulted in a significant increase of about 78% in the mean neurite length per cell, when compared with the GRG formulation only (Table 3 and Figure 1). The mean neurite length per cell following treatment with GRG was 260.77 ± 40.68 vs. 449.13 ± 31.66 μm per cell following treatment with AGRG (Figure 1 and Table 3; *p* < 0.05, using one-way ANOVA followed by Dunnett's test). Analyzing the total number of neurites shows that AGRG treatment resulted in an increase of proximally 4-fold vs. GRG treatment (186 vs. 46, respectively). Treatment with higher concentrations of Copaxone did not result in further increase in total neurite growth. The number of neurons also showed an increasing trend following AGRG treatment (GRG: 14; AGRG: 64). The number of neurites per cell and the number of bifurcation per cell were not significantly different when comparing the GRG treatment vs. the AGRG treatment. Interestingly, treatment with Copaxone alone (without GRG), at a concentration of 10 μg/ml, showed an increase of 66.02% in the mean neurite length per cell, compared with the GRG formulation only (data not shown).

**Table 3:** Effects of AGRG different formulations on neurite outgrowth.

**Treatment**	**Total number of neurites**	**Total number of cells**	**Mean neurite length/cell (μm)**	**Mean number of neurites/cell**	**Mean number of bifurcations/cell**
DMEM	15	7	107.38 ± 27.16	2.08 ± 0.27	0.33 ± 0.33
GRG	46	14	260.77 ± 40.68	3.29 ± 0.34	2.13 ± 0.70
AGRG	64	186	449.13 ± 31.66^*^	2.96 ± 0.18	1.92 ± 0.23

* *p < 0.05 vs. GRG, using one-way ANOVA followed by Dunnet's test*.

### *In vivo* Studies

Following finalization of the GRG and AGRG formulations in an *in vitro* assay, we decided to conduct an *in vivo* study on rats to see which tocopherol concentrate ion is most efficient. Thus, we conducted an acute PNI model in rats, with a nerve deficit of 10 mm. The rats were treated as described in Table 1 and were followed up for a period of 5 months. During this period, the rats underwent electrophysiological assessments, clinical scoring, and functional recovery.

**Figure 2:**
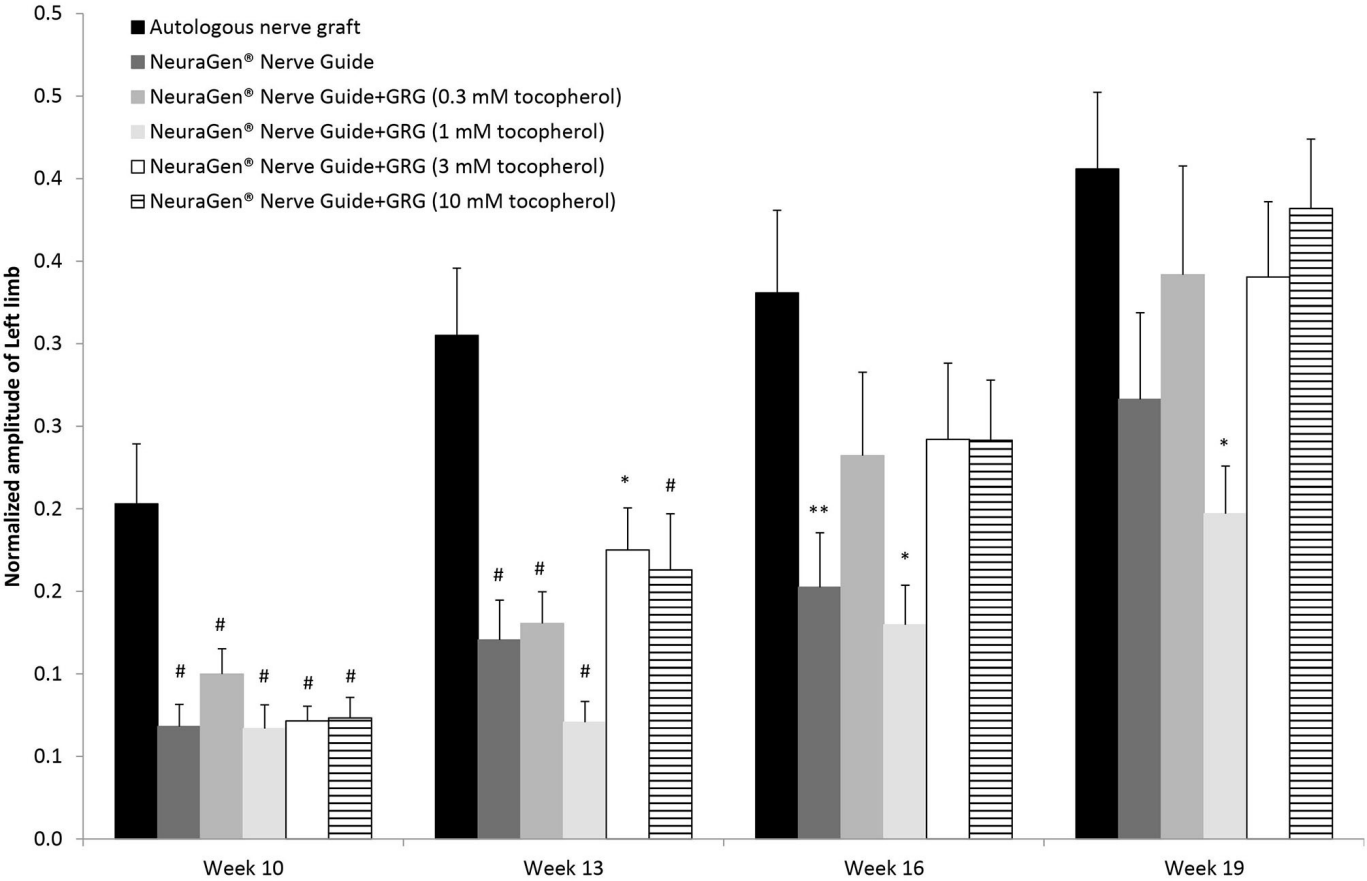
CAMP normalized amplitude. The CMAPs amplitude values of the left limb measured at weeks 10, 13, 16 and 19 weeks after surgery, were normalized to the amplitude measured at baseline. Results are presented as mean ± SEM. Asterisk represents statistical significance: *p < 0.05 vs. autologous nerve graft, using one-way ANOVA followed by Tukey's HSD test; **p < 0.05 vs. autologous nerve graft, using one-way ANOVA followed by Holms test; ^#^*p*< 0.01 vs. autologous nerve graft, using one-way ANOVA followed by Tukey HSD test.

Figure 2 displays the left hind limb's normalized amplitude (to baseline) of the CMAPs measured from the tibialis anterior muscle. Generally, the normalized amplitude values increased for the injured left hind limb during the entire study period. Ten weeks after surgery, we observed a slight recovery in all groups; in the ANG group, the recovery was significantly higher (0.20 ± 0.04; *p* < 0.01, using one-way ANOVA followed by Tukey HSD test). Toward the end of the study, on week 16, the normalized amplitude of the ANG treatment was 0.33 ± 0.05, significantly higher than the normalized amplitude of the NeuraGen® Nerve Guide tube (0.15 ± 0.03; *p* < 0.05, using one-way ANOVA followed by Holms test), although this difference was not detectable at week 19. Treatment with GRG containing 1 mM tocopherol showed significantly lower normalized amplitude when compared with the ANG treatment [*p* < 0.01 (week 13) and *p* < 0.05 (weeks 16 and 19), using one-way ANOVA followed by Tukey HSD test]. From week 16, there is no significant difference between the ANG treatment and treatment with GRG containing 0.1, 3, or 10 mM tocopherol. These findings suggest that treatments with GRG containing 0.1, 3, and 10 mM tocopherol are as beneficial as the ANG treatment, repairing a 10-mm gap of the sciatic nerve.

**Figure 3:**
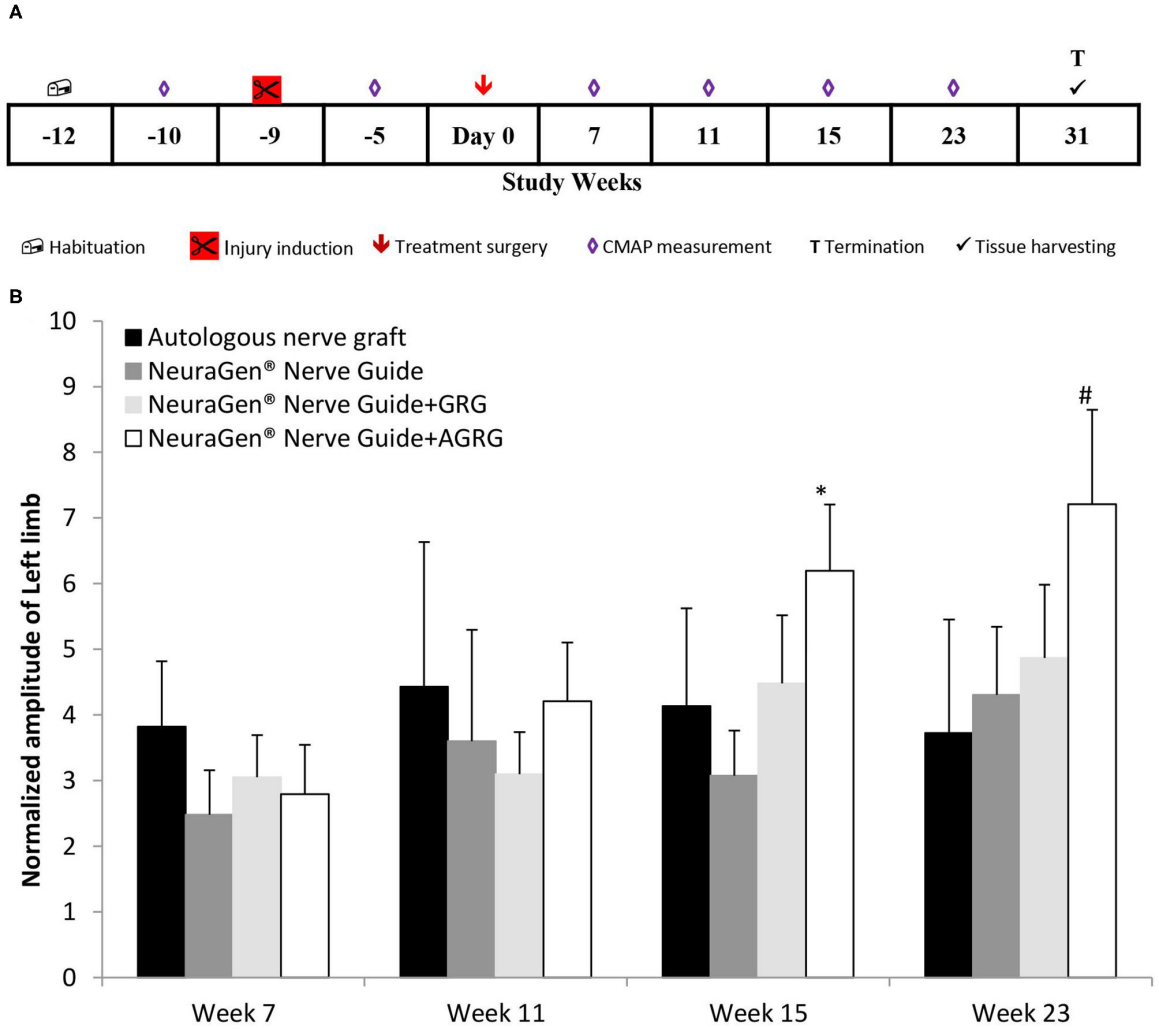
Chronic PNI model. **(A)** Study design. The injury was induces 9 weeks prior the treatment surgery and the rabbits were followed up for 31 weeks post treatment. **(B)** Left limb (injured) CAMP normalized amplitude. The amplitude values of the CMAPs measured at weeks 7, 11, 15 and 23 weeks after treatment, were normalized to the amplitude measured 5 weeks prior the treatment surgery (after the injury inducing and before the treatment surgery). Results are presented as mean ± SEM. Asterisk represents statistical significance: *p < 0.05 vs. NeuraGen® Nerve Guide, using one-way ANOVA followed by Dunnett's test; ^#^*p* < 0.05 vs. week 7, using one-way ANOVA followed by Tukey HSD test.

Combining the findings of both the *in vitro* and the *in vivo* studies, we set the tocopherol concentration of 3 mM on the GRG formula. Then, we conducted a chronic PNI model on rabbits with a critical gap of 25 mm in the tibial portion of the sciatic nerve, to assess the effect of the GRG and AGRG hydrogels on nerve reconstruction. The rabbits were treated as described in Table 2 and were followed up for a period of 31 weeks after treatment (Figure 3A). During this period, the rats underwent electrophysiological assessments (until week 23; Figure 3B) and clinical scoring.

Following transection of the tibial portion of the sciatic nerve and preservation of the peroneal portion, the CMAPs were measured from the gastrocnemius muscle. The signal during the entire study in the injured limb (left) was markedly lower than that of the right limb throughout the study, with an exception of the ANG treatment, in which the right hind limb was also injured (data not shown). However, observing the normalized amplitude of the left limb to the amplitudes values measured between surgeries (week−5; Figure 3A), the treatment with NeuraGen® Nerve Guide+AGRG show a significant higher value than that of the NeuraGen® Nerve Guide, at week 15 (Figure 3B; 6.20 ± 1.01 vs. 3.08 ± 0.69, respectively; *p* < 0.05, using one-way ANOVA followed by Dunnett's test). This finding was also observed at week 23, when NeuraGen® Nerve Guide+AGRG showed the highest result in comparison to the other treatments. Although this finding is not statistically significant at week 23, the trend continues to show that AGRG treatment is the most promising for reconstruction of the tibial portion of the sciatic nerve with critical gap of 25 mm. It is important to emphasize that the baseline values of the CMAPs, measured from both hind limbs, were within the normal range (data not shown; right hind limb: 17.24 ± 0.88 mV; left hind limb: 18.06 ± 0.90 mV).

Upon harvest the tibial portion of the sciatic nerve for immunohistochemistry analysis at weeks post-treatment, we assessed the quality of the samples by preforming H&E staining. The H&E staining showed that most proximal cross sections were unaffected or contained a mild vacuolization of the nerve's fibers and a very mild lymphocytic infiltration. In the distal sections were mostly mildly affected with fibers vacuolization (data not shown).

**Figure 4:**
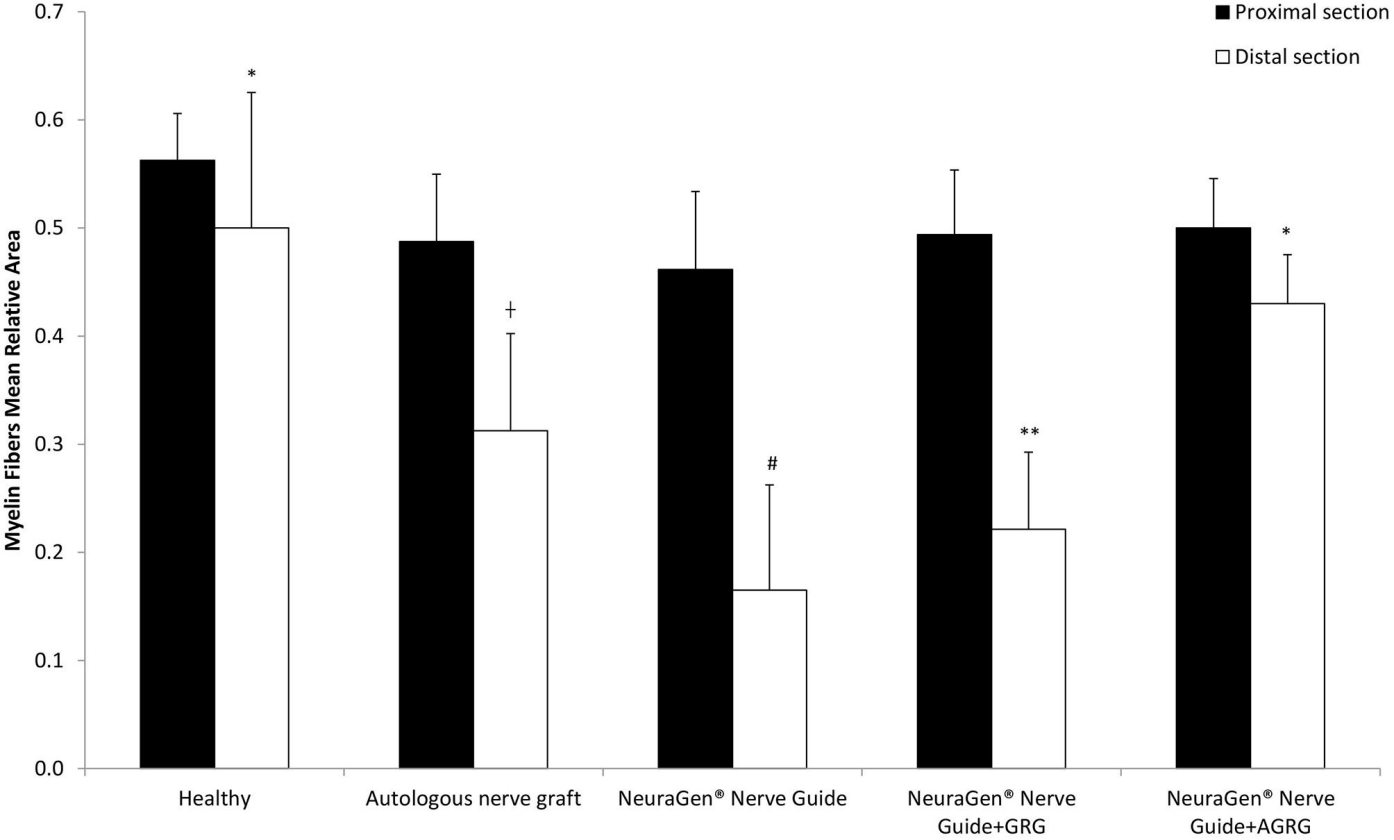
MBP staining to assess regeneration. **(A)** The graph display the mean relative area (mean ± SEM) of the MBP in Prox and Dist sections. Asterisk represents statistical significance: *p < 0.1 using two-tailed Student's *T*-test vs. NeuraGen® Nerve Guide; **p < 0.01 using two-tailed Student's *T*-test vs. proximal section; ^#^*p* < 0.05 using two-tailed Student's *T*-test vs. proximal section; ^†^*p* < 0.1 using one-tailed Student's *T*-test vs. proximal section.

**Figure 5:**
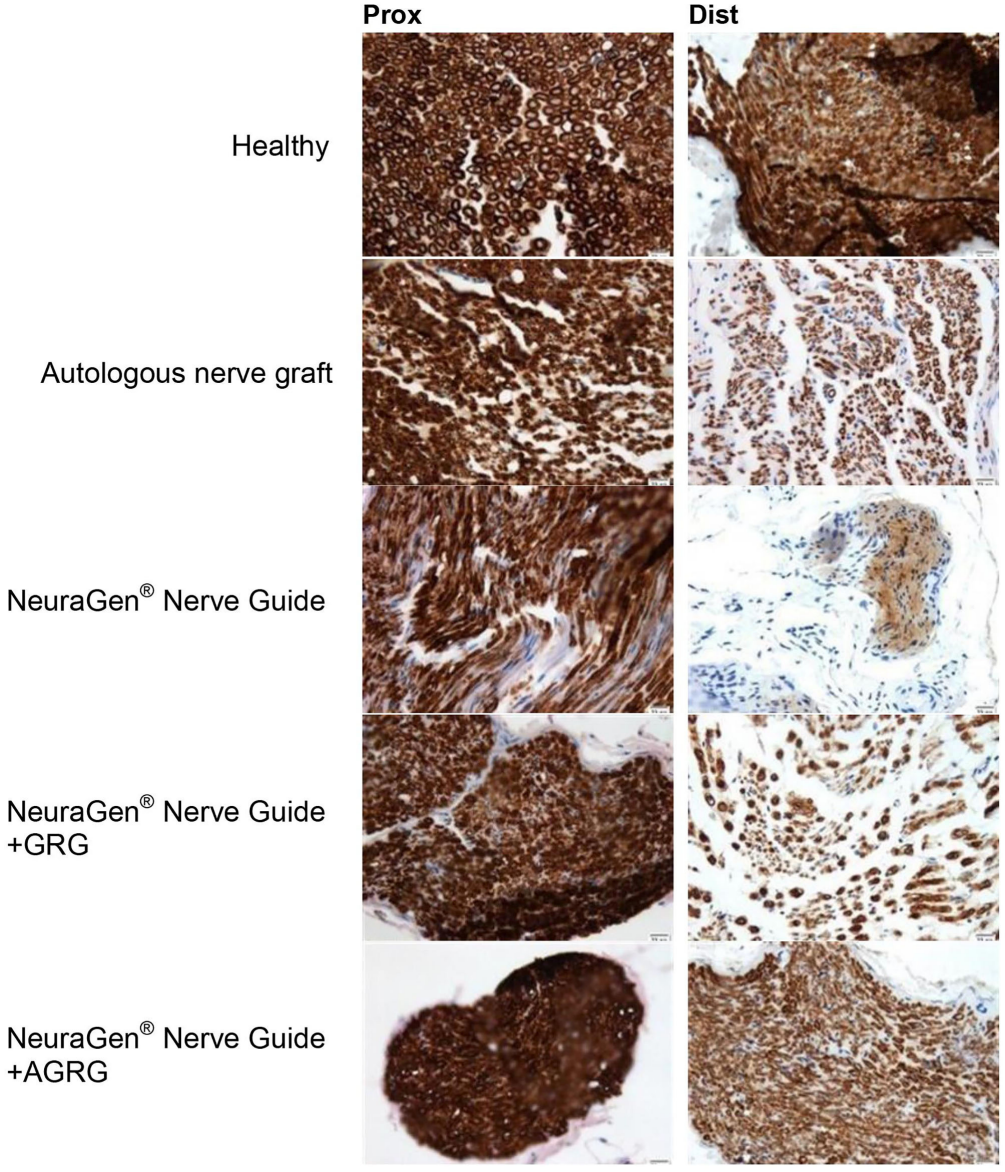
Representative histologic pictures of the MBP staining. The proximal and distal sections of the tibial portion of the sciatic nerve of each treatment are displayed. Pictures were taken at magnification of ×20.

Then, we stained the samples with myelin-based protein (MBP) to evaluate the nerve reconstruction. The MBP mean relative area values of the proximal sections are similar, as the healthy section, regardless the treatment (Figures 4, 5 and Table 4). When observing the regeneration process of the distal sections (Figures 4, 5 and Table 4), there is a significant regeneration of the AGRG treatment, compared with the NeuraGen® Nerve Guide treatment (Figures 4, 5 and Table 4; ^*^ *p* < 0.1, two-tailed Student's *T-*test).

**Table 4:** MBP assessment.

**Treatment**	**Prox**	**Dist**
Healthy	0.56 ± 0.04	0.50 ± 0.13^*^
Autologous nerve graft	0.49 ± 0.06	0.31 ± 0.09^+^
NeuraGen® Nerve Guide	0.46 ± 0.07	0.17 ± 0.10^#^
NeuraGen® Nerve Guide filled with GRG	0.49 ± 0.06	0.22 ± 0.07^**^
NeuraGen® Nerve Guide filled with AGRG	0.50 ± 0.05	0.43 ± 0.05^*^

The values of the MBP mean relative area are presented as mean ± SEM. Asterisk represents statistical significance:

* p < 0.1 using two-tailed Student's T-test vs. NeuraGen® Nerve Guide;

** p < 0.01 using two-tailed Student's T-test vs. proximal section;

# p < 0.05 using two-tailed Student's T-test vs. proximal section;

+ *p < 0.1 using one-tailed Student's T-test vs. proximal section*.

According to the distal sections findings we can conclude that the AGRG treatment succeeded to significantly regenerate the injured lesion, as good as the ANG and healthy groups, after 31 weeks post-treatment months from treatment; while the NeuraGen® Nerve Guide treatment show a mild regeneration process.

## Discussion

The ultimate goal of present study on GRG/AGRG is to improve the functional performance and quality of life of patients affected by PNI with massive loss defect, which represents a major cause for morbidity and disability in affected patients and may cause substantial costs for the society in a global perspective.

The current clinical gold standard for peripheral nerve reconstruction, when larger nerve gaps exist (20 mm or longer in humans), is an autologous sensitive nerve graft (autograft). The reconstruction of a segmental nerve loss poses a significant surgical challenge in order to achieve better results and lower donor morbidity. Reinnervation with cutaneous sensitive nerves is not always satisfactory, as motor fibers need to be included into the bridging nerve grafts (19). In addition, nerve harvesting and subsequent donor site morbidity lead to functional loss, as well as to an increased risk of neuroma formation, paresthesias, and higher costs associated with a second surgical site (20). Moreover, long nerve gap lengths have been among the most difficult injuries to repair, demonstrating slow rates of regeneration and often incomplete recovery. Thus, further development of novel concepts to accommodate longer nerve deficits must be encouraged.

One of the promising solutions already in clinical practice is artificial nerve conduits. The most significant advantage to using commercial nerve conduits is to avoid sacrificing the patient's functional nerve for an autograft. The procedure is simpler, there is a significant decrease in time of surgery, and there is no sensation loss or cosmetic defect in the leg. These are the advantages of using nerve conduits in comparison to ANG, explaining the efforts invested in optimizing this solution worldwide. Experimental research with simple nerve guiding conduits showed unsuccessful bridging of relatively long gaps of 15 mm in the rat (21), of ~30 mm in rabbits, and of 30 mm in primates (22–24). Results from clinical studies are often comparable to autografts in the treatment of lesions with nerve defects of <3 cm. These models do not assure nerve regeneration in more extensive lesions. Therefore, the disadvantage of commercial nerve conduits is the inability to bridge more than 2–3-cm-long nerve loss. Another methodology is based on a decellularized cadaveric nerve (allograft), which is prepared through a process of detergent decellularization, enzyme degradation, and gamma irradiation sterilization (25). Acellular nerve allografts rather than fresh allografts do not need immunosuppression and appear to be effective based on clinical studies (26). The decellularization methods reported in the literature give rise to a series of disadvantages, such as an increased risk of contamination, technical incompatibility (27), and compromised tissue functionality after gamma-ray sterilization (28). Other attempts to improve nerve regeneration is developed with conduit luminal scaffolds, from collagen and laminin hydrogels to synthetic and collagen filaments and channels (20, 29–33). However, these modifications have not produced results better than those of the autograft and therefore do not offer a substantial benefit over the autograft at this time (31, 34, 35).

Although nerve conduit has advantages, in comparison to the ANG, the nerve conduit's inability to bridge a gap of over 2–3 cm of nerve loss prevents its widespread application in clinical practice for reconstruction of peripheral nerves. Therefore, the repair and regeneration of peripheral nerve injuries with massive loss defects still remain a major clinical issue for the relatively new fields of regenerative medicine and biomaterials and tissue engineering.

We started to investigate the possibility of increasing nerve regeneration through a long-distance gap by using a composite neurotube in 2004 (36) and created a GRG matrix (8) that would serve as a vehicle to axonal growth and surviving and therefore enable the reconstruction of peripheral nerves with massive loss defect.

Our current study suggests that the modified procedure of using a commercial nerve conduit filled with a newly developed AGRG formulation for nerve reconstruction may be successfully used in clinical practice for treatment of PNI with massive loss defect. We base our statement on the positive effect we received in the treatment of a rabbit model of delayed (chronic) PNI that represents the most common human condition of delayed PNI with a gap of more than 2 cm. We used delayed nerve repair because in clinical practice, it often occurs and is indicated in complex cases of severe local soft tissue and/or bony injuries associated with a significant area of nerve injury and a ragged nerve transection (37).

In the present study, GRG formulation was modified, and a novel combination of AGRG prepared with FDA-approved agents was investigated *in vitro* on the neuronal outgrowth. Application of AGRG (GRG+Copaxone) showed a significant increase of about 78% in neurite length per cell and was shown to have the most promising effect on neuronal outgrowth (Figure 1 and Table 3). In addition, the total number of neurites increases by 4-fold when compared with the GRG formulation only.

For the finalization of the GRG and AGRG formulations, different concentrations were added in an *in vitro* assay. We decided to conduct an *in vivo* study on rats to investigate which tocopherol concentration is most efficient. We found that the GRG+tocopherol treatments are as beneficial as the 10-mm ANG. Then, we conducted a study on a rabbit model of delayed (chronic) PNI with a critical gap of 25 mm (a model that imitated the human condition of delayed repair and large gap) to assess the effect of the GRG and AGRG hydrogels on nerve recovery. Nine weeks after injury, the nerve was repaired.

The electrophysiological follow-up showed that AGRG treatment is the most promising for reconstruction of the tibial portion of the sciatic nerve with a critical gap of 25 mm (Figure 3). Moreover, a surprising finding was the beneficial effect of AGRG when compared with the autologous nerve reconstruction.

Thirty-one weeks post the second surgery (delayed reconstruction), histochemical observation showed significant regeneration after using AGRG hydrogel, compared with the empty tube (Figures 4, 5 and Table 4). Based on to the distal sections findings, we can conclude that the AGRG treatment succeeded to significant nerve regeneration nerve, as well as the ANG and healthy groups.

*In conclusion*, we demonstrate that in our injury model of a delayed nerve repair with massive nerve loss defect, the application of AGRG led to a stronger nerve recovery than other reconstructive strategies in the past.

## Data Availability Statement

The raw data supporting the conclusions of this article will be made available by the authors, without undue reservation.

## Ethics Statement

The animal study was reviewed and approved by Council for Experiments of Animal Subjects at the Israeli Ministry of Health.

## Author Contributions

SR co-invented the matrix, conceived and designed, planned the experiments, and surgery. MA planned the experiments and conducted the electrophysiological assessment. SM conducted the *in vitro* and *in vivo* experiments and data analysis. ZN co-invented the matrix. All authors contributed to the article and approved the submitted version.

## Conflict of Interest

The authors declare that this study received funding from Baxter International. The funder had the following involvement with the study: study design and supervision.
